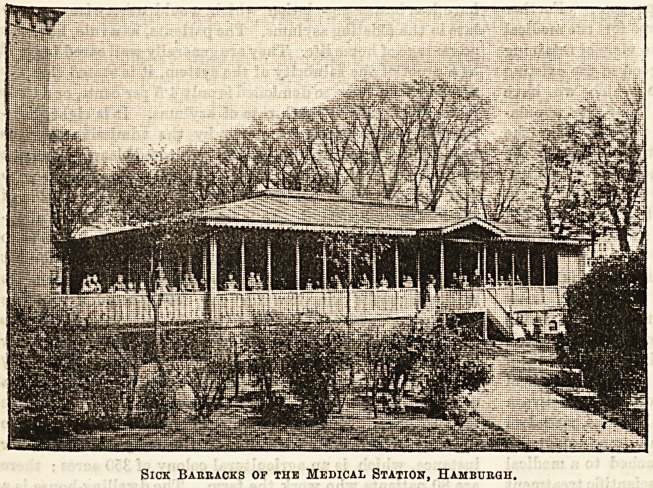# "Barraque" Wards

**Published:** 1892-07-09

**Authors:** 


### HOSPITAL CONSTRUCTION.

### "BARRAQUE" WARDS.

The extreme importance of fresh air as a curative agent, more particularly in surgical cases, is nowadays pretty widely recognised, and it is interesting to observe the developments to which the necessity for obtaining a plentiful supply gives rise. In some hospitals—as, for example, the Berlin Town Hospital (Freidrichshrain)—covered balconies are provided opening out of the wards into which the patients are wheeled in their beds. In others, wooden buildings are erected in the garden, having their sides largely open, there being no windows but only curtains, which can be drawn at night, or to protect the patients from the glare of the sun. One such ward, or "barraque" as they are called, is shown in the illustration, which is reproduced from a photograph of the original at Hamburg. The structure is entirely of wood, and is freely open at the sides. The patients, therefore, while protected from rain, and excessive heat are, to all intents and purposes, in the open air. Wards of this kind are to be seen in many foreign hospitals. In the old and, in many respects, defective hospital at Basel there is an excellent baraque ward in the garden, in which children with surgical ailments are kept night and day for as long a time as the weather permits. On an average this period lasts from May until September, both months included.

Sick Barracks of the Medical Station, Hamburgh.

## Figures and Tables

**Figure f1:**